# Tat-specific antibodies associated with better HIV-associated motor function

**DOI:** 10.1038/s41598-025-12624-0

**Published:** 2025-08-11

**Authors:** Catarina E. Hioe, Clauvis Kunkeng Yengo, Xiaomei Liu, Reuben Davis, Gospel Enyindah-Asonye, Jéromine Klingler, Alex F. Tang, Juan C. Bandres, Anuradha Ganesan, Tahaniyat Lalani, Joseph Yabes, Brian K. Agan, Xiaokun Liu, David J. Volsky, Susan Morgello, Jessica Robinson-Papp

**Affiliations:** 1 James J. Peters VA Medical Center, Bronx, NY USA; 2 Department of Medicine, Division of Infectious Diseases, Icahn School of Medicine at Mount Sinai, New York, NY USA; 3 Division of Infectious Diseases, Walter Reed National Military Medical Center, Bethesda, MD USA; 4 Infectious Disease Clinical Research Program, Department of Preventive Medicine and Biostatistics, Uniformed Services University of the Health Sciences, Bethesda, MD USA; 5 Henry M. Jackson Foundation for the Advancement of Military Medicine, Inc., Bethesda, MD USA; 6 Division of Infectious Diseases, Naval Medical Center Portsmouth, Portsmouth, VA USA; 7 Division of Infectious Diseases, Brooke Army Medical Center, San Antonio, TX USA; 8 Department of Neurology, Icahn School of Medicine at Mount Sinai, New York, NY USA

**Keywords:** HIV, Tat, Antibodies, Epitopes, Dopaminergic neuron, Neuropathogenesis, Immunology, HIV infections, Medical research, Preclinical research

## Abstract

Tat is a transactivator of HIV transcription that can be secreted to affect nearby cells. Deleterious central nervous system effects of extracellular Tat include dopaminergic neuron dysfunction, raising the possibility that Tat secretion may be linked to Parkinsonian motor features seen in people with HIV (PWH). This study examined the potential association of anti-Tat antibodies and extrapyramidal motor dysfunction in PWH. In 42 PWH who were chronically infected and characterized for neurological abnormalities, anti-Tat IgG levels were lower in individuals with more severe extrapyramidal motor abnormalities as measured by the Unified Parkinson’s Disease Rating Scale (UPDRS). The association was observed specifically with IgG against the cysteine-rich Tat region. However, the IgG responses to Tat were weak in comparison to responses against the other HIV antigens. This disparity was evident in plasma and cerebrospinal fluid, and was also seen in early infection, before antiretroviral therapy. When the effects of antibodies on Tat function were evaluated, PWH plasma had no activity against Tat-induced transactivation, while Tat-immunized mouse and rabbit sera exhibited a potent inhibitory activity. These data provide initial evidence for a protective potential of anti-Tat antibodies and raise the possibility that a therapeutic Tat vaccine might alleviate clinical signs of HIV-associated dopaminergic dysfunction.

**Supplementary Information:**

The online version contains supplementary material available at 10.1038/s41598-025-12624-0.

## Introduction

People with HIV (PWH) have high rates of neurological complications, including a range of behavioral, motor, and cognitive dysfunctions^1,2^. With highly potent combination antiretroviral therapy (ART), severe brain disorders such as dementia have become rare and milder phenotypes of neurocognitive impairment have become more common. PWH on ART also continue to manifest subtle motor abnormalities. A longitudinal analysis of 78 ART-treated PWH enrolled in the Manhattan HIV Brain Bank revealed declining motor function during ≥ four years of follow-up, with increasing scores on the HIV Motor Scale (HMS), while cognitive impairment remained mild and stable^3^. Notably, HMS ratings were strongly associated with the motor section of the Unified Parkinson’s Disease Rating Scale (mUPDRS), a widely used measure of the extrapyramidal motor and gait abnormalities seen in Parkinson’s disease (PD)^4,5^. HIV infection has also been shown to cause neuropathology in multiple dopaminergic regions of the central nervous system (CNS) in ART-naïve and ART-treated PWH^6–8^. Alterations in dopaminergic neurotransmission, including a substantial reduction of dopamine levels in the substantia nigra−the main site of dopamine production in the CNS−are a key factor correlated with the development of HIV-associated neurological disorder^6,9^although CNS glutamatergic and cholinergic systems have also been implicated^10–14^.

The mechanisms involved in the progression of HIV-associated motor deficits are not well defined. HIV primarily infects macrophages and microglial cells in the brain; these cells serve as virus reservoirs and secrete viral proteins, such as Tat (transactivator of transcription) and gp120, as well as inflammatory cytokines and chemokines, which together are believed to cause neuronal damage and neurological disease^15^. HIV Tat, in particular, has been shown to impair the structure and function of dopaminergic neuronal cells^9^. Notably, as much as 65% of Tat produced by infected cells is secreted via a distinct leaderless secretory pathway and this extracellular Tat can be taken up by neighboring uninfected cells^9,16–18^. In the serum of PWH, the amount of Tat is around 0.1 nM, similar to the levels in the supernatants of HIV-infected cell cultures^19,20^. Tat persists in the blood of ART-treated PWH at levels independent of CD4 T cell counts or viral loads, indicating continual Tat production despite immune restoration and virus control by ART^21^. Moreover, low level HIV replication was shown in the CNS of PWH on ART^22–27^ and the Tat transcript was among those detected in the brain of fully suppressed ART-treated PWH^28^. In a cross-sectional study of PWH on long-term ART, Tat protein was detected in CSF samples from 36.8% of these individuals, while in longitudinal CSF analysis, 66.7% of PWH were positive for Tat at one or more time points post-ART^29^. In this study, the presence of Tat protein in CSF was strongly associated with psychostimulant drug use and poor psychomotor speed and information processing performance^29^.

Many studies have examined the effects of Tat protein on neuronal and non-neuronal cells present in the CNS. Tat-induced calcium release was associated with neurotoxicity in primary human neuronal cells in vitro^30^. Through its cysteine-rich and core regions, Tat also affected monocyte chemotaxis^19^ and modulated inflammatory cytokine production in primary human monocytes and astrocytes^31^. In rat models, administration of Tat alone either by a Tat-expressing SV40 delivery vector in the brain or by injecting Tat protein to the caudate putamen−the brain region involved in the dopamine nigrostriatal transmission pathway−resulted in neuronal apoptosis and oxidative stress^32^. Furthermore, treatment of dopaminergic rat cells with Tat or Tat cDNA inhibited tyrosine hydroxylase (TH), a rate-limiting enzyme in the dopamine biosynthetic pathway, and reduced dopamine production and release^33^. Correspondingly, injection of Tat protein into the striatum induced a subclinical Parkinson’s-like disease that was marked by a reduced staining of TH-expressing neurons^33^. Results from a mouse model expressing Tat under doxycycline control further confirmed alterations of TH expression in distinct brain regions of the dopamine pathways^34^. In addition, recently studies pointed to a direct interaction of Tat with human dopamine transporter to affect the transporter conformational transition and inhibit the dopamine reuptake activity critical for dopamine homeostasis^35–37^providing another mechanism for Tat-induced perturbation of dopamine neurotransmission. Lastly, the synergistic effects of Tat and psychostimulant drugs that target the dopaminergic system, such as cocaine and methamphetamine, have been demonstrated in vitro and in animal models^9^.

There is yet no effective treatment to alleviate or cure HIV-associated neurological impairment. In view of the significant role of the HIV Tat protein in promoting neuronal dysfunctions, targeting Tat with antibodies or vaccines is an idea to consider for treating neurological complications of HIV. When antibodies specific for Tat were detected in CSF of PWH, the levels were notably higher in PWH with normal cognitive functions than in PWH with mild to moderate cognitive impairment, although they were also higher in PWH with higher viral load and lower CD4 cell counts^38^. In contrast, the anti-Tat antibody responses induced by a Tat protein vaccine tested in ART-treated PWH in phase II clinical trials were associated with increasing CD4 T cell count and decreasing proviral DNA^39–41^. This vaccine was designed as an adjunctive therapy for ART intensification; the vaccine effect on neurological and other morbidities associated with chronic HIV infection was not studied.

In this study, we sought to examine the protective potential of antibodies against Tat in PWH presenting with varying types and degrees of neurological abnormalities. The levels of plasma IgG antibodies specific for Tat and the six defined regions of Tat were evaluated and correlated with the neurological test scores. The data demonstrated an inverse correlation between the IgG responses to Tat, specifically the dominant cysteine-rich region of Tat, and the severity of extrapyramidal motor abnormalities. However, anti-Tat antibody responses induced naturally during HIV infection were relatively weak and did not exert measurable Tat-inhibitory activities in vitro, although potent Tat-neutralizing antibodies could be generated in animals upon vaccination. Data from this study implicate the protective potential of anti-Tat antibodies and proffer the utility of a Tat vaccine to elicit such antibodies to alleviate motor abnormalities in PWH.

## Results

### The levels of anti-Tat antibodies were lower in PWH with the highest range of mUPDRS scores indicative of more severe extrapyramidal motor abnormalities

Because of the documented role of Tat in neuronal toxicity and dysfunction, we examined whether anti-Tat antibodies might potentially exert a protective effect. To this end, the relative levels of plasma IgG antibodies against Tat and three other HIV antigens (gp120, p24 and Nef) were compared in 42 PWH from the Manhattan HIV Brain Bank Study. These 42 individuals were evaluated for neurological abnormalities, which were quantified by the mUPDRS, a measurement that includes motor and gait abnormalities associated with dopaminergic neuronal dysfunctions as found in patients with PD^4,5^ and by the results of a comprehensive neurocognitive testing battery summarized as a Global T-score, an overall measure reflective of neurocognitive function encompassing the following domains: motor, speed of information processing, working memory, memory encoding, memory retrieval, language fluency, and abstraction/executive function^42,43^ (Supplemental Table S1).

These participants (median age = 60.5; range = 51–72) were on ART for 1 to 18 years (median = 7.4 years). Viral load measurements were collected throughout the study period and used to calculate the areas under the curve (AUC). The annualized viral loads (AUC/year) were found to range from undetectable to 4.7 × 10^4^ copies (Supplemental Table S1). CD4 T cell counts at the time of sample collection also varied from 25 to 1394 (median = 546), with 12 PWH having CD4 counts below 350. CD4 count negatively correlated with viral load AUC/year (*r* = −0.38, *p* = 0.013 by Spearman correlation). Age did not correlate with CD4 T cell counts nor with viral loads (*r* = 0.031, *p* = 0.848 and *r* = −0.081 *p* = 0.611, respectively, by Spearman correlation).

**Fig. 1:**
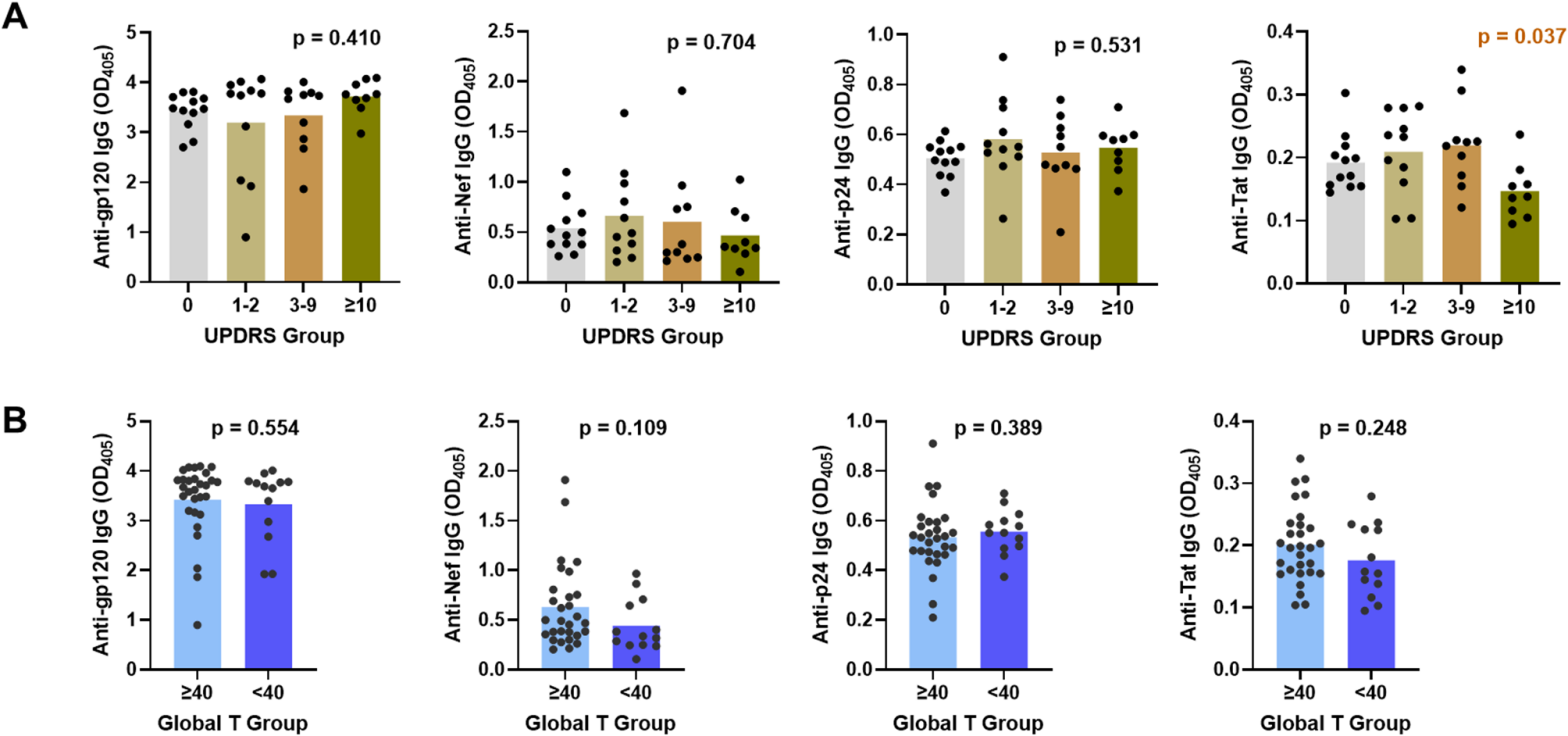
Levels of antibodies against Tat in PWH with more severe extrapyramidal motor abnormalities as measured by UPDRS scores of ≥ 10. The levels of plasma antibodies to gp120, Nef, p24, and Tat were examined in ELISA using a colorimetric substrate to yield optical density readings at 405 nm (OD_405_). **A **Relative levels of plasma IgG antibodies against the four HIV antigens in PWH with varying UPDRS scores. The 42 PWH were grouped into four quartiles based on their UPDRS scores. p values from ANOVA are shown. **B** Relative IgG levels against the same four antigens in PWH with Global T-scores of ≥ 40 (normal) or < 40 (deficient). p values are from Mann-Whitney test.

Plasma samples from 42 PWH were obtained at the same day of the neurological examinations and evaluated in ELISA for IgG against gp120, Nef, p24, and Tat. Control plasma samples from five persons without HIV (PWOH) were tested in parallel. A one-way ANOVA test was performed to compare IgG antibody levels against each HIV antigen in four quartiles of PWH on the basis of mUPDRS. A significant difference was detected for anti-Tat antibodies among PWH with different mUPDRS scores, whereas no significant differences were observed with antibodies against the three other HIV antigens (Fig. 1A). The plot further shows an inverse association between anti-Tat antibodies and mUPDRS scores. In particular, the levels of anti-Tat IgG were lower in PWH with more severe abnormalities (mUPDRS scores of 10 or greater), but similar in PWH with normal (mUPDRS score of 0) and moderate scores (mUPDRS scores of 1–9), suggesting a potential contribution of anti-Tat antibodies against the more severe PD-like disorders in PWH. Age, CD4 T cell counts, and viral loads did not differ among the four quartiles (Supplemental Fig. S1). By contrast, no differences were apparent in antibody levels against Tat and the other tested antigens in PWH with normal (≥ 40) or impaired (< 40) Global T-scores (Fig. 1B).

### Lower levels of antibodies against the dominant cysteine-rich region of Tat were also found in PWH with more severe extrapyramidal motor abnormalities

**Fig. 2:**
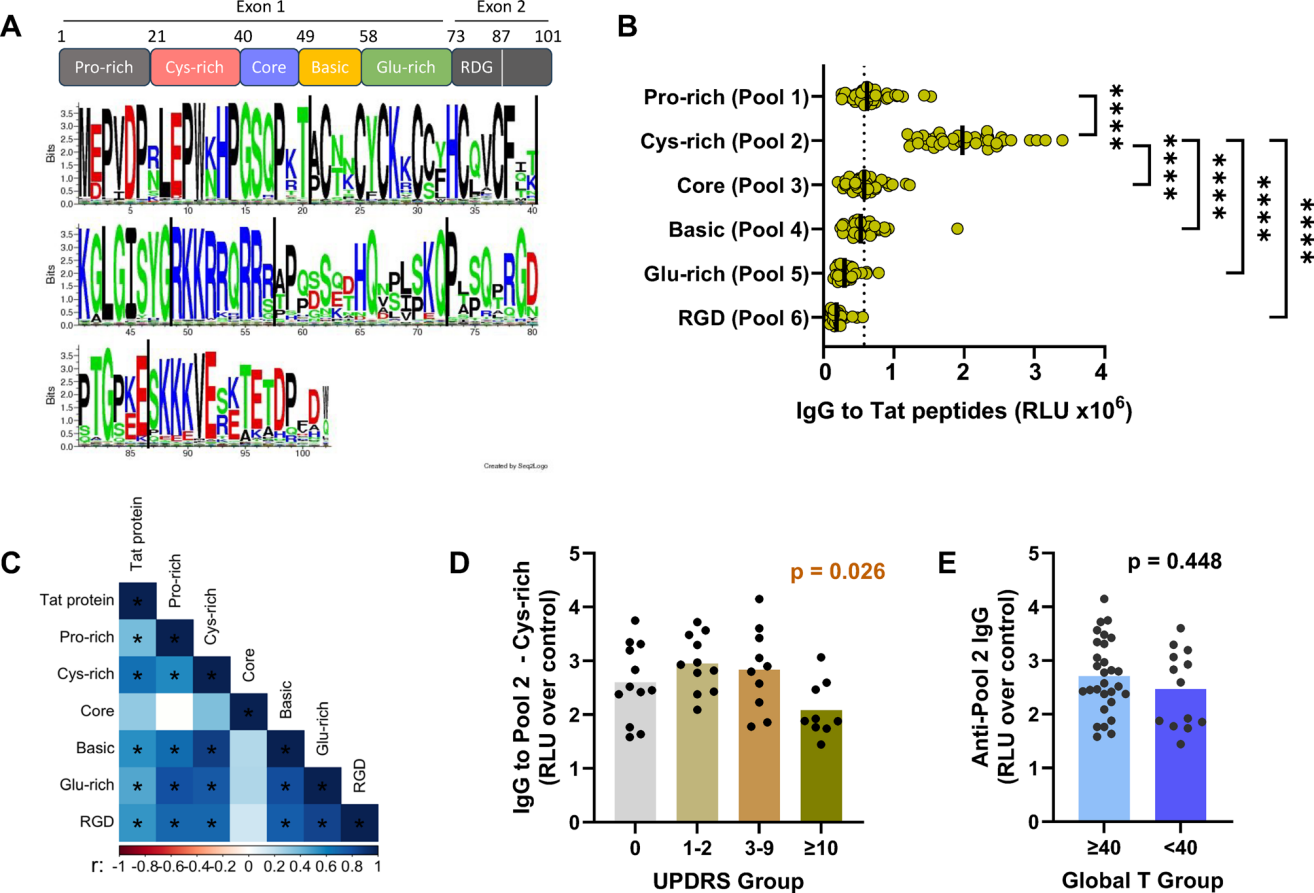
Lower levels of IgG antibodies against the dominant cysteine-rich region of Tat in PWH with more severe mUPDRS scores of ≥ 10. Six peptide pools were tested in ELISA for IgG reactivity with plasma (1:100) from 42 PWH. Binding IgG was detected using alkaline phosphate-conjugated secondary antibodies and a luminescent substrate that provided a broader range of signal (relative luminescent units or RLUs). Five PWOH controls were tested in parallel; the mean RLU is depicted by the dotted line in Panel B. **A ** A diagram to illustrate the six regions within the 101 amino acid-long Tat protein encoded by two exons and a logo of HIV-1 Tat sequences from the Los Alamos Database to show the conserved motifs and variability in the different regions. **B** IgG reactivity of PWH plasma with six pools of overlapping 15-mer Tat peptides. The Tat peptide sequences are shown in Supplementary Fig. S2. **** *p* < 0.0001 by ANOVA; only comparisons with pool 2 are shown. **C** Correlation matrix of Ig responses to Tat protein and peptide pools. Spearman r values are color coded; p values are marked with * if *p* < 0.05 or otherwise left unmarked. **D** Relative levels of plasma IgG antibodies against pool 2 in four groups of PWH based on their UPDRS scores. p values from ANOVA are shown. Data for the other peptide pools are shown in Supplemental Fig. S3A. **E** Relative levels of plasma IgG against pool 2 in PWH with Global T-scores of ≥ 40 (normal) or < 40 (impaired). p values from Mann-Whitney test are shown. Data for the other peptide pools are shown in Supplemental Fig. S3B.

HIV Tat protein is encoded by two exons and made of six regions with relatively conserved proline-rich, cysteine-rich, core and basic regions in the N-terminal segment and more variable glutamine-rich and integrin-binding RGD regions toward the C-terminus (Fig. 2 A). To define the regions targeted by anti-Tat antibodies generated in PWH, plasma samples from the 42 PWH were tested for IgG reactivity against six pools of overlapping 15-mer peptides representing the six regions of Tat (Fig. 2A and Supplemental Fig. S2). The IgG responses to the N-terminal regions were relatively strong in comparison with the responses to the C-terminal regions, and the highest responses were against the cysteine-rich region (pool 2) (Fig. 2B). The magnitude of IgG reactivity to the Tat protein and peptide pools positively correlated with each other, except for the responses to core peptides (pool 3) which showed no correlation with any others (Fig. 2 C). The strongest correlation was for IgG reactivity to Tat protein vs. the cysteine-rich pool 2 peptides (*r* = 0.658, *p* = 2.2 × 10^−6^ by Spearman correlation), in line with the cysteine-rich region as the dominant target of the anti-Tat IgG responses.

Among the four quartiles of mUPDRS scores, PWH with more severe mUPDRS scores of ≥ 10 had lower levels of IgG antibodies to the highly conserved cysteine-rich region of Tat (Fig. 2D), which has been shown to mediate some of Tat’s pathogenic activities^44–48^. No such association was observed with IgG against other Tat regions (Supplemental Fig. S3A). By contrast, the IgG levels against the cysteine-rich region did not differ among PWH with normal versus impaired global T-scores (Fig. 2E). Similarly, no differences were observed with IgG against the other Tat regions in the two groups (Supplemental Fig. S3B). 

### In contrast to the robust responses to Env, Nef, and Gag, minimal antibody responses were elicited against Tat during early and chronic HIV infection

**Fig. 3:**
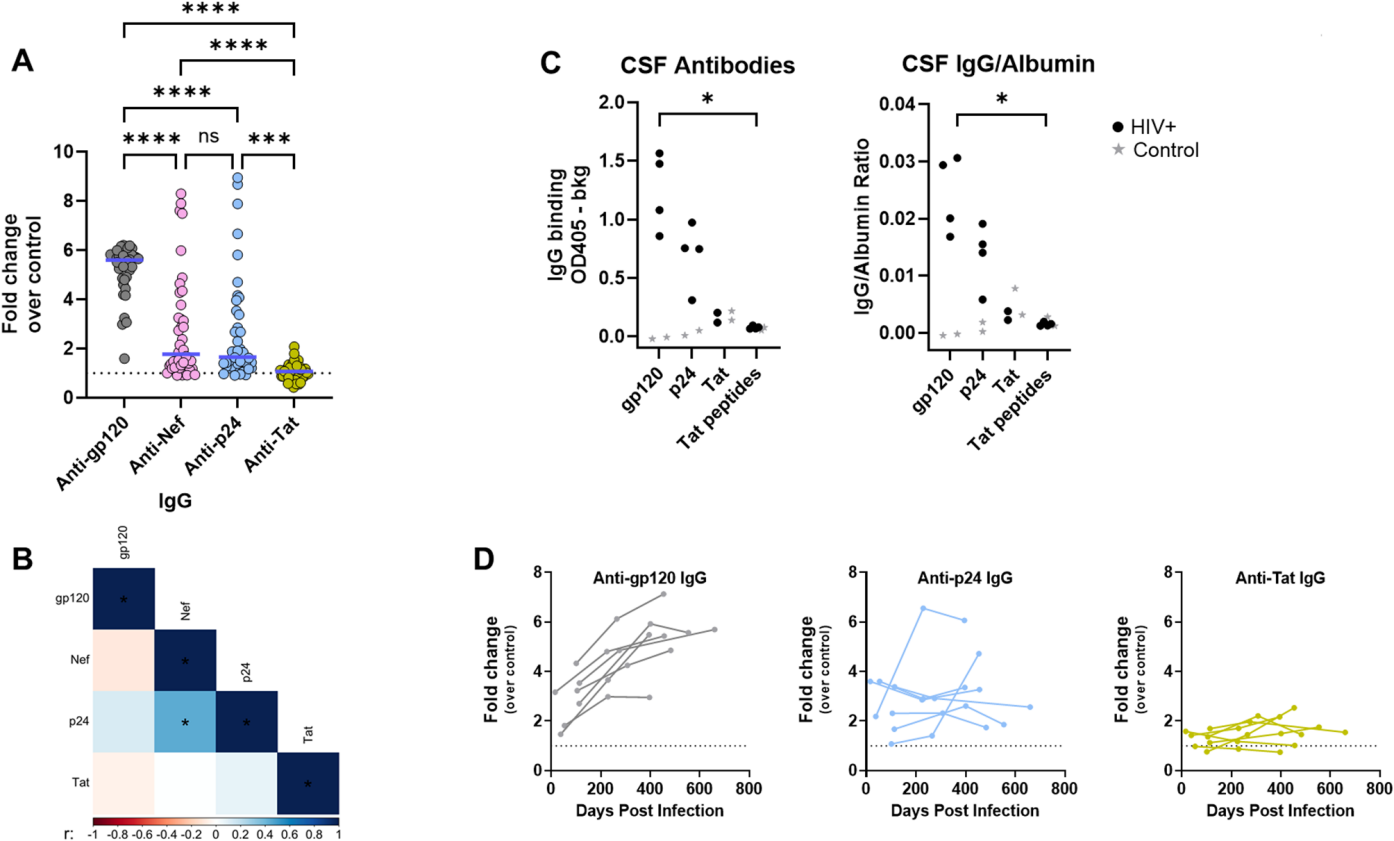
In contrast to the robust responses to Env, Nef, and Gag, minimal anti-Tat antibody responses were detected in serum and CSF samples from PWH during chronic or early infection. ELISA was performed to detect IgG antibodies against Env gp120, Nef, Gag p24, and Tat as described in Fig. 1. Plasma and CSF samples were tested at 1:100. **A** Relative levels of plasma antibodies against gp120, Nef, p24, and Tat generated during chronic HIV infection in 42 PWH plasma samples. Fold changes were calculated over the mean of five PWOH controls (set to 1, dotted line). *** *p* < 0.001; **** *p* < 0.0001; ns *p* > 0.05 by ANOVA. **B** Spearman correlation of antibody responses against the four HIV antigens in the 42 PWH samples. Correlation coefficients (r) are color coded; * denotes *p* < 0.05; *p* ≥ 0.05 are unmarked. **C** Detection of antibodies against p24 and gp120, but not Tat, in the PWH CSF samples as indicated by ELISA OD_405_ values (left panel) and the anti-HIV IgG/albumin ratios (right panel). CSF IgG against gp120, p24, Tat protein and Tat peptides were evaluated in samples from four PWH and two PWOH. The four PWH had high levels of plasma antibodies to p24 and gp120 (1.7- to 14-fold change over control) and very low Tat-specific plasma antibodies (1.1- to 5.0-fold changes over control). The albumin concentrations in CFS were 48–54 µg/mL for PWH and 28–44 µg/mL for PWOH. * *p* < 0.05, *p* > 0.05 unmarked by mixed effects models on PWH data points only. **D** Relative levels of IgG antibodies against gp120, p24, and Tat in seven PWH at three time points during early infection prior to ART.

Anti-Tat IgG responses observed in Figs. 1–2 were relatively weak. To compare IgG responses to the different HIV antigens, the fold changes over the respective controls were calculated (Fig. 3A**)**. IgG responses to Tat were indeed lower than those to gp120, Nef, and p24. The responses to gp120 were the most robust with almost all PWH showing > four-fold changes over control (median = 5.6). Anti-Nef and p24 IgG levels were not as strong (median fold changes of 1.8 and 1.7, respectively) and more variable (ranging from 0.9 to 9). By contrast, the antibody responses to Tat were uniformly weak (median = 1.1). They were undetectable in 18 PWH, and the remaining 24 PWH had minimal responses that were slightly above control. Notably, the magnitude of IgG responses against the four antigens did not correlate with each other, except for a weak but significant correlation between anti-Nef and anti-p24 antibody levels (Fig. 3B).

To evaluate whether antibodies against HIV were present in the CNS, we tested cerebrospinal fluid (CSF) specimens available from four of the PWH studied here and two PWOH for control. The four PWH had low-level plasma IgG against Tat and high-level plasma IgG to p24, and gp120, approximating the entire PWH group (Fig. 3A). The levels of IgG antibodies in CSF reflected those in plasma. Anti-Tat IgG was not evident in CSF, whereas antibodies against p24 and gp120 were readily detected above control in each of the four CSF samples (Fig. 3C left panel). The pattern was similar for the ratios of virus-specific IgG/albumin concentrations (Fig. 3C right panel), as the albumin concentrations were comparable for CSF samples from PWH (48–54 µg/mL) and PWOH (28–44 µg/mL), indicating the presence of virus-specific IgG in CSF of PWH without reduced integrity of the blood brain barrier.

Subsequently, the IgG responses to gp120, p24, and Tat were examined in PWH during early infection before ART initiation. Plasma samples were collected at three time points prior to ART from seven PWH who enrolled in the U.S. Military HIV Natural History Study^49^ (age range = 20–29 years at the initial time point) (Fig. 3D). The time points ranged from 16 to 660 days (median = 265 days) from the estimated time of HIV exposure. Plasma viral loads were detected at each of the three time points for all seven PWH (log_10_ vRNA range = 3.45 to 5.84) (Supplemental Table S2). The ELISA data showed poor induction of IgG responses to Tat at early times after infection even when virus replication was not controlled with ART (Fig. 3D). In contrast, anti-gp120 IgG antibodies were clearly detectable in all samples and the levels increased over the three time points tested. p24-specific IgG antibodies were also evident for all seven PWH; the levels increased for two PWH and did not change over time for the other five. However, anti-Tat IgG responses were low or similar to control and the levels were stably below two-fold over control. Altogether, these data demonstrate that weak IgG responses were elicited against Tat throughout early HIV infection prior to ART and such low responses were also observed during the chronic infection.

### Plasma antibodies in PWH were insufficient to block Tat-mediated activities, although Tat-neutralizing antibodies could be generated by immunizing rabbits and mice

**Fig. 4:**
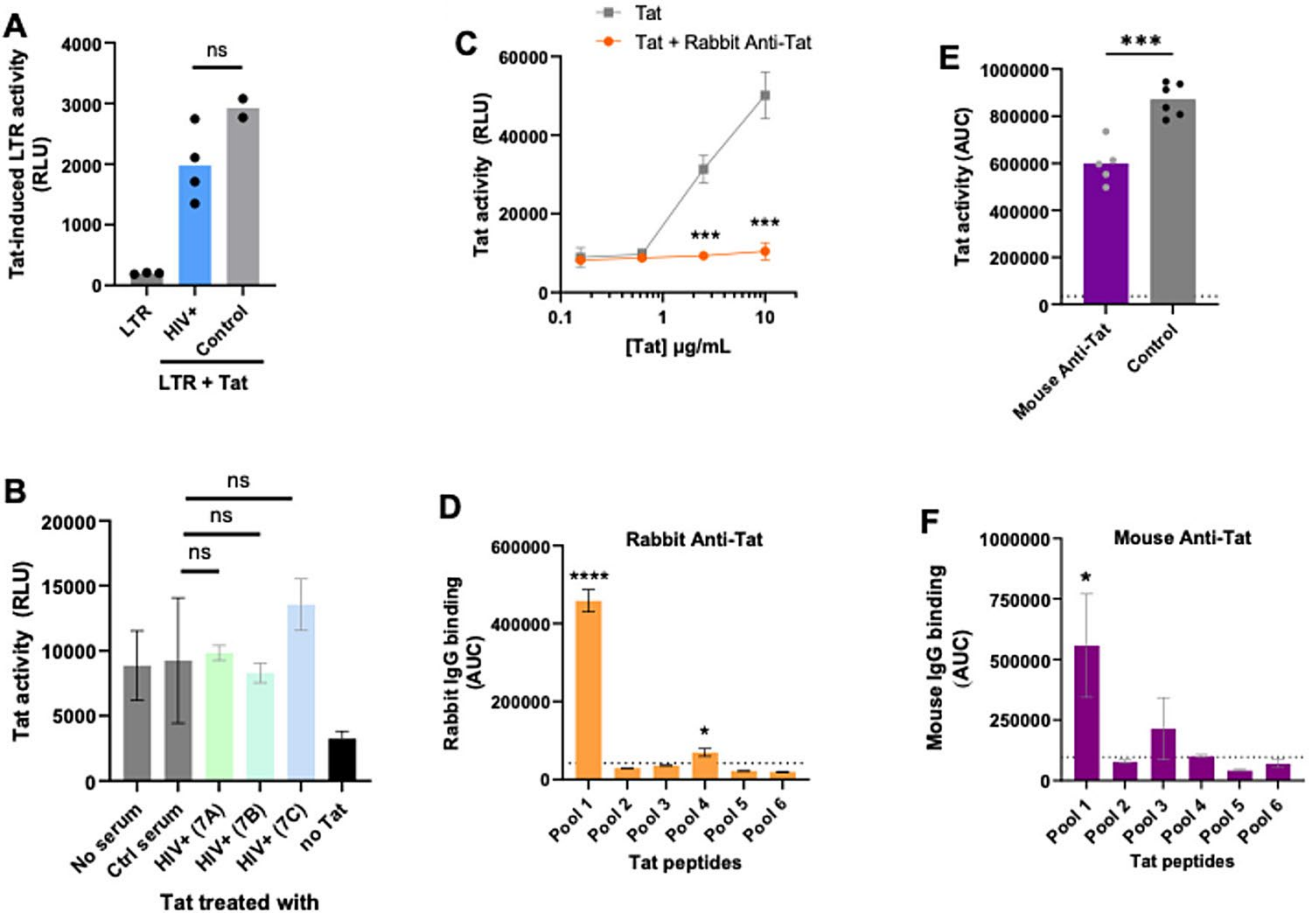
Unlike anti-Tat serum from immunized rabbits and mice, plasma antibodies from PWH were insufficient to inhibit Tat transactivation. **A** PWH sera displayed weak or no inhibitory effects on Tat activity. Tat activity was measured using Tat-expressing 293 T cells co-cultured with 293 T cells transfected with an HIV-1 L-controlled luciferase gene (pGL3-LTR-luc). Serum samples (1:20) from PWH on ART or PWOH (control) were incubated for 4 h with Tat-expressing 293 T cells prior to co-culture. Luciferase activity was measured in the co-cultures after an overnight incubation. LTR: background activity without luciferase. Each condition was tested in triplicates and experiment was performed twice. Mean and standard deviations from one experiment are shown. ns *p* > 0.05 by t test. **B** PWH sera also did not block the activity of recombinant Tat protein. Tat protein (5 µg/mL) was treated with PWH sera from early infection prior to ART or control PWOH sera (1:10) and then incubated with TZM.bl cells bearing a Tat-inducible luciferase reporter gene. Cells treated with Tat alone or no Tat were included as positive and negative controls, respectively. Luciferase activity was measured after 48 h. Each condition was tested in triplicates. ns *p* > 0.05 by ANOVA. **C** Serum from Tat-immunized rabbits was effective in blocking Tat activity. Titrating amounts of recombinant Tat protein was pre-incubated with immune rabbit serum (1:10) or medium and added to TZM.bl cells. Luciferase activity was measured as described in panel B. *** *p* = 0.0004 vs. untreated Tat. **D** Serum antibodies from Tat-immunized rabbits were reactive most strongly with the proline-rich region (pool 1) of Tat. Immunization was performed with Tat 1–61, which includes only the four N-terminal regions corresponding to pools 1 to 4. Serially diluted serum was tested for IgG binding to six pools of Tat peptides as described in Fig. 2. Area under the titration curve (AUC) for each peptide pool is shown. The titration curves are shown in Supplemental Fig. S4D. **** *p* < 0.00001; * *p* < 0.05 vs. control (dotted line) by ANOVA. **E** Serum samples from Tat-immunized mice were also effective in blocking virus-derived Tat activity. Titrated sera from mice immunized with a Tat-expressing LNP-mRNA vaccine were incubated with virus and TZM.bl cells. Tat-induced luciferase activity was measured after 48 h. *** *p* < 0.001 vs. control mice by t test; dotted line: background. **F** Serum IgG from Tat-immunized mice also recognized mainly Tat peptides encompassing the proline-rich region (pool 1). Mice were immunized with Tat 1–86, which includes regions 1–5 and part of region 6. Titration curves are shown in Supplemental Fig. S4E. * *p* < 0.05 vs. control (dotted line) by ANOVA.

To examine the inhibitory activity of antibodies against Tat, serum samples from PWH were tested in two assays that measured Tat activities using the luciferase reporter under the HIV LTR control. In the first assay, Tat-transfected 293 T cells were incubated with plasma samples from four PWH or two PWOH, and co-cultured with 293 T cells transfected with the LTR-luciferase reporter (pGL3-LTR-luc). The LTR-luciferase activity was detected in the Tat and LTR-Luc co-cultures but not in the co-cultures without Tat. A trend of reduced Tat-induced luciferase activity was seen upon treatment with PWH versus control plasma, but the inhibitory levels were not significant (Fig. 4A).

In the second assay, a recombinant Tat protein was used to elicit Tat-inducible luciferase activity in the TZM.bl reporter cells. Pre-incubation of Tat protein with plasma samples from three PWH also did not affect Tat activity (Fig. 4B). On the other hand, rabbit anti-Tat serum showed strong neutralizing activity against Tat (Fig. 4C). The antiserum was raised against Tat 1–61 encompassing the amino terminal segment of Tat from the proline-rich region to the basic region and had a Tat-specific IgG titer much higher than those of PWH plasma samples (Supplemental Fig. S4A-B). The peptide epitope mapping also showed a distinct profile from that of PWH plasma (Fig. 4D, Fig. 2B). The rabbit IgG reacted mainly to the proline-rich region (pool 1) and, at a lower extent, the basic region (pool 4) (Fig. 4D, Supplemental Fig. S4D). Similar results were observed with mice immunized with a lipid nanoparticle (LNP)-encapsulated mRNA vaccine expressing Tat 1–86, except that the mouse IgG recognized primarily pool 1 peptides and reacted weakly and variably with pool 3 peptides and not pool 4 peptides (Fig. 4E-F, Supplemental Fig. S4C and E). For both rabbit and mouse antibodies, the titers to Tat protein (end-point > 10^5^) were higher than those to pool 1 peptides (end-point ~ 10^3^−10^4^), suggesting the involvement of antibodies against non-linear conformational Tat epitopes. These data provide preliminary evidence that the weak anti-Tat antibody responses elicited during early or chronic HIV infection had minimal inhibitory activity, but high-level anti-Tat responses with potent neutralizing activity and distinct peptide specificity, could be elicited by vaccination.

## Discussion

In this study on ART-treated PWH presenting with a spectrum of neurologic dysfunction, we found an inverse association of anti-Tat antibody levels with the severity of extrapyramidal motor dysfunction (typically due to dopaminergic neuronal dysfunction) but not with neurocognitive dysfunction. These results are in line with accumulating data from in vitro studies and in animal models documenting the detrimental effects of Tat on dopaminergic neuronal structures and functions^9,32–37,50^. In addition, Tat along with gp120 has been reported to contribute to glutamate excitotoxicity by reducing glutamate uptake while stimulating its release from macrophage/microglial cells and over activating the receptors, thus increasing CSF extracellular glutamate that promotes neuronal toxicity^51,52^. Nonetheless, antibody responses to Tat were minimally generated in PWH with chronic infection after ART initiation and this was observed similarly in both plasma and CSF specimens. In contrast, strong IgG responses were elicited to the other HIV proteins such as gp120, p24, and Nef, indicating that the poor responses against Tat were specific and not associated with overall immune suppression. Similarly low or undetectable levels of anti-Tat antibody responses were detected during early infection prior to ART, a disease stage characterized by high level virus replication. These findings are consistent with past data demonstrating that < 50% of PWH were positive for anti-Tat antibodies^53^ and when detected the anti-Tat responses were weak throughout two years after HIV seroconversion^54^. Despite their low magnitudes, the presence of anti-Tat antibodies was associated with slower disease progression, greater CD4 T cell counts, CD4/CD8 T cell ratios, and lower viral burden in several independent studies^55–58^indicative of the protective potential of anti-Tat antibodies.

The mechanistic explanation for the weak induction of anti-Tat antibodies during HIV infection remains unclear. However, the failure of PWH to mount robust responses against Tat during acute and chronic HIV infection is not a reflection of the inherent Tat immunogenic potential. When administered as protein or DNA vaccines in different formulations, Tat readily elicited anti-Tat antibody responses in animals^59–63^. The vast majority of human volunteers with or without HIV immunized with Tat protein also generated Tat-specific IgM, IgG, and IgA responses that persisted for many years^39–41,64,65^. Anti-Tat antibodies elicited in ART-treated PWH after Tat vaccination exhibited the capacity to inhibit Tat-induced HIV replication and Env entry into dendritic cells in vitro^39,41,64^. The vaccine-induced immune responses were also associated with improvements in immune preservation or restoration and proviral DNA decay^40,41,64,65^demonstrating the capacity of Tat vaccination to elicit functional anti-Tat antibodies effective against the virus. However, these trials were not conducted in older PWH who are at risk of HIV-induced neurologic impairments and thus the effects on Tat vaccination on HIV-associated neurological dysfunctions remain to be determined. Moreover, a vaccine strategy focusing only on Tat may not be sufficient, as other viral proteins such as Env, Vpr, Vpu, and Nef also demonstrate neurotoxic properties^66–70^. A vaccine to prevent or alleviate HIV-induced neurologic dysfunctions is likely to require a composite of immunogens that can elicit robust inhibitory antibody responses against all neuropathogenic antigens.

Our Tat epitope mapping using overlapping consensus B Tat peptides demonstrated a superior recognition of the cysteine-rich region over the other five Tat regions by serum antibodies from ART-treated PWH evaluated in this study. The serum IgG reactivity against the intact Tat protein and the cysteine-rich peptides also correlated strongly. Although the magnitude of IgG responses against these Tat antigens was very low, the data indicated the cysteine-rich region as the dominant target of antibodies raised by PWH from our US cohort. A similar finding was reported in PWH cohorts in southern India^71^. In this earlier study, the peptide scan analysis defined the cysteine-rich domain of the clade C Tat to be the most immunodominant region. Interestingly, antibodies against the clade C Tat protein were cross-reactive with Tat of clades D and AE, but not as much with Tat of clades A and B. Nonetheless, the same pattern of antibody reactivity across the different Tat regions was observed and the same dominant region was identified for clades C and B Tat antigens. Antibodies against the cysteine-rich region were also found to be effective in blocking Tat-induced HIV transactivation^71^. With seven conserved cysteine residues, this region of Tat was shown to be critical for Tat-mediated neuronal pathologic effects, as exemplified by inhibition of neprilysin, a neuronal endopeptidase responsible for Aβ degradation in the brain^45^inhibition of the neuroprotective Wnt/β-catenin pathway in astrocytes^44^and induction of synaptodendritic injury in primary hippocampal neurons^47^. However, it remains unclear if antibodies against the cysteine-rich region of Tat can provide protection against HIV-associated motor disorder. This study presents only correlative data that lower IgG levels against the cysteine-rich domain were associated with more severe motor dysfunction, especially in PWH with mUPDRS of > 10. Further studies are required to assess whether antibodies against the cysteine-rich region indeed have protective effects against HIV-induced motor impairment.

Interestingly, anti-Tat antibodies raised by vaccination did not target the cysteine-rich region. Rather, the dominant responses were against the proline-rich region. These data were observed similarly in rabbits and mice immunized with a Tat protein and a Tat mRNA vaccine, respectively. In human volunteers who received a Tat protein vaccine, Tat peptide-specific IgG and IgM responses were also predominantly against the proline-rich region^64^. These vaccine-elicited responses exhibited Tat-inhibitory activity, indicating the protective potential of antibodies against the proline-rich region. In PWH, antibody responses to the proline-rich region were very weak, thus it is yet undetermined whether high titers of antibodies to this region would help ameliorate HIV-associated motor dysfunction. Further studies are also needed to test different Tat vaccines for the ability to generate antibody responses to target a broader range of epitopes including those in the cysteine-rich and other regions of Tat. Of note, the truncated Tat 1–61 and Tat 1–86 proteins were used to vaccinate animals in this study and human volunteers in the past study^64^. Future investigation into the magnitude and breadth of anti-Tat responses to different vaccine formats or formulations is warranted for the development of more efficacious Tat vaccines capable of eliciting potent neutralizing antibodies against the various activities of Tat across different strains and clades.

There are several limitations in this study. The numbers of PWH evaluated were relatively small and the analyses of PWH subgroups based on UPDRS were especially underpowered, limiting the generalizability of the findings. Only cross-sectional samples were studied from these PWH subgroups, without longitudinal follow-ups, precluding any inference whether anti-Tat responses indeed mitigate the severity of neurodegeneration over time. However, the study included a unique cohort of older PWH participants enrolled in the Manhattan HIV Brain Bank. Because this exceptional group of PWH presented a wide spectrum of comprehensively phenotyped motor and cognitive disorders, we were able to identify for the first time an inverse association between the severity of extrapyramidal motor dysfunction measurable by mUPDRS and the levels of IgG antibodies specifically against HIV Tat, thus suggesting the notion of protective effects of anti-Tat antibodies against dopaminergic neuronal deficits in older PWH. Nonetheless, this exploratory study presented only correlative data and lacked functional validation for the role of anti-Tat antibodies in neuroprotection. The evaluation of Tat-specific antibodies was also very limited. Because of the limited sample amounts, we did not enrich for Tat-specific IgG from plasma of PWH to measure inhibitory activities that might be detectable at higher concentrations. Moreover, the CSF antibody levels were correlated only with those of albumin as an indicator of blood brain barrier integrity; no other markers of CNS injury or inflammation were evaluated. Vaccination of rabbits with Tat 1–61 did not yield antibodies against the cysteine-rich region; thus, the functionality of antibodies against this region remains unknown. The Tat-specific antibodies raised during HIV infection and by vaccination in rabbits and PWH^64^ might also be directed to conformational epitopes that could not be defined by short peptides. In addition, future investigation is needed to explore the Tat immunogens and vaccine delivery systems capable of eliciting potent neutralizing antibodies broadly reactive with Tat from the myriad of circulating HIV strains and effective against the Tat regions associated with neurotoxicity and other pathogenic properties.

## Materials and methods

### Human specimens

Plasma samples were obtained from 42 ART-treated PWH who were enrolled in the Manhattan HIV Brain Bank (MHBB, PI: Morgello), an ongoing longitudinal cohort study. Participants were consented for the use of their samples in research, utilizing forms and protocols approved by the Icahn School of Medicine at Mount Sinai (ISMMS) Institutional Review Board (IRB) (Protocol #:11–00388). As per MHBB procedures, all participants underwent a standardized neurologic examination (inclusive of the mUPDRS) performed by a neurologist and comprehensive neurocognitive testing; these procedures have been previously described^72^. The resulting scores and other relevant clinical information are listed in Supplemental Table S1. We also collected negative-control plasma samples from PWOH enrolled in the James J. Peters Veterans Affairs Medical Center (IRB Protocol #: BAN-1604).

In addition, we examined longitudinal plasma samples from participants of the U.S. Military HIV Natural History Study conducted by the Infectious Disease Clinical Research Program (IDCRP) at the Uniformed Services University of the Health Sciences within the US Department of Defense (IRB Protocol #: IDCRP-000-05)^49^. These samples were collected from seven PWH at three time points prior to ART initiation, and the estimated periods post HIV exposure for all time points ranged from 16 to 660 days (median: 265 days). The CD4 T cell count, virus load, and demographic data are compiled in Supplemental Table S2.

All participants were over 18 years old, signed written informed consent, and provided permission for sample banking and sharing. The study was performed according to the approved IRB protocol at each institution in compliance with the principles of the Declaration of Helsinki. All samples were heat inactivated before use in the assays.

### HIV proteins and peptides

HIV-1 JRFL gp120 protein was purchased from Immune Technology (Catalog #: IT-001-0024p). The following reagents were obtained through the NIH HIV Reagent Program, Division of AIDS, NIAID, NIH: Tat protein from HIV-1 IIIB, recombinant produced in E. coli, HRP-2222; HIV-1 consensus B Tat peptide set (Catalog #: 5138); HIV-1 HXB2 p24 recombinant protein, ARP-13,126; HIV-1 HXB2 Nef recombinant protein, ARP-13,342; all contributed by DAIDS, NIAID.

### Mouse immunization

BALB/c mice (*n* = 5, female, 6 weeks old from the Jackson Lab) were immunized with an LNP-mRNA vaccine expressing NL4.3 Tat 1–86 (GenBank AF324493.2). After four monthly intramuscular injections (10 µg/dose), terminal blood was collected and tested in the study. The mRNA vaccine was produced using the SM-102 LNP formulation by the ISMMS RNA NanoCore of the Icahn Genomics Institute. Animal procedures were approved by the ISMMS Institutional Animal Care and Use Committee and performed in accordance with the approved protocol, and the authors complied with the ARRIVE guidelines.

### ELISA to measure plasma IgG against HIV antigens

ELISA was performed as described in^73^. Briefly, diluted plasma or serum was incubated with each antigen that was pre-coated overnight on ELISA plates (2 µg/mL). IgG reactivity was measured using alkaline phosphatase-conjugated anti-human IgG secondary polyclonal antibodies (Southern Biotech, Catalog #: 2040-04), followed with the colorimetric PNPP substrate (ThermoFisher, Catalog #: 34047) or KPL PhosphaGLO luminescent substrate (SeraCare, Catalog #: 5430-0055). To detect rabbit and mouse IgG, secondary polyclonal antibodies against IgG of the relevant species were used (SouthernBiotech, Catalog#: 4055-04 and Abcam, Catalog#: AB97020). For ELISA with pooled peptides, the peptide mixtures were prepared with an equal concentration of each peptide and coated on ELISA plates at 1 µg/mL of each peptide.

### Tat transactivation assays

The first assay was performed with Tat-expressing 293 T cells upon transient transfection with plasmid pcDNA-flag-tat_NDK_ (0.3 µg per well in 24-well plates) after 24 h. After washing and incubation for another 4 h, the cells were co-cultured at a 1:1 ratio with 293 T cells previously transfected with pGL3-LTR-luc (0.1 µg per well in 24-well plates). After an overnight co-culture, the cells were lyzed and luciferase activity was measured. In the second assay, Tat protein was pre-treated with diluted plasma samples from PWH or PWOH or with rabbit anti-Tat serum and incubated for 48 h with the TZM.bl reporter cells bearing a Tat-inducible luciferase gene. The following reagent was obtained through the NIH HIV Reagent Program, Division of AIDS, NIAID, NIH: Polyclonal Anti-HIV-1 Tat Protein (antiserum, rabbit), ARP-705, contributed by Dr. Bryan Cullen. The third assay was performed using virus-derived Tat expressed by HIV-1 pseudovirus with TH023.6 Env to test mouse anti-Tat serum samples. Diluted sera were added to virus and incubated with TZM.bl cells for 48 h, and luciferase activity was measured on cell lysates described as above.

### Statistical analyses

Correlation, t test, or ANOVA were performed as designated in the figure legends using GraphPad Prism 10. Correlation matrices were generated using R (version 4.2.3, the R Foundation for Statistical Computing) and corrplot package.

## Electronic supplementary material

Below is the link to the electronic supplementary material.

Supplementary Material 1

Supplementary Material 2

Supplementary Material 3

## Data Availability

Data reported in this paper are presented in the supplemental tables. Additional data will be shared upon request to the corresponding author.
