# Supplementary material for: Tat-specific antibodies associated with better HIV-associated motor function
Source: Sci Rep. 2025 Aug 11;15:29353. doi: 10.1038/s41598-025-12624-0 (PMC12339940; doi:10.1038/s41598-025-12624-0)
Supplement: Supplementary file 3 — Supplementary Material 3 [file 41598_2025_12624_MOESM3_ESM.pdf]

Figure S1

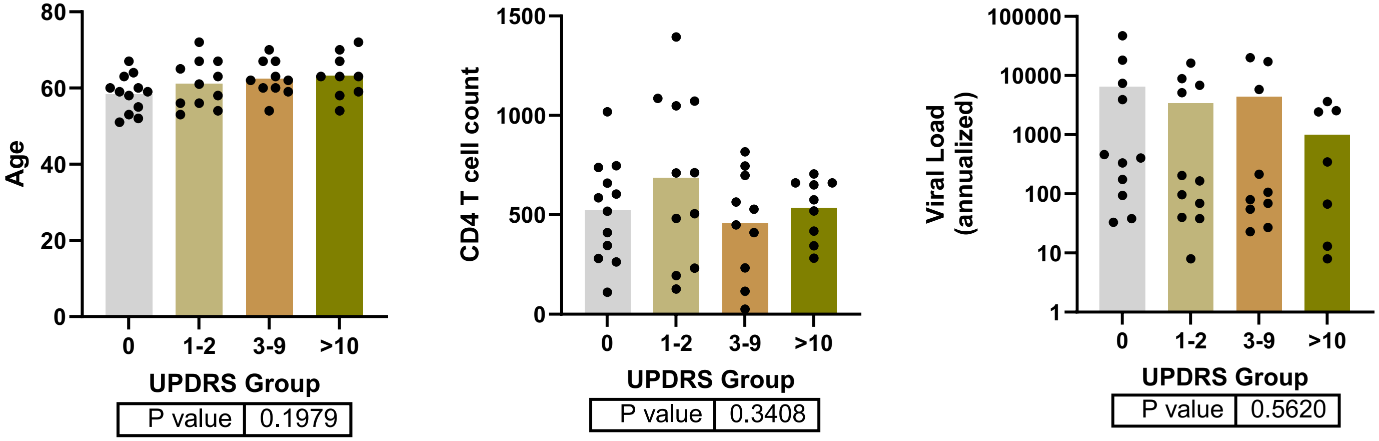


**Supplemental Figure S1. The UPDRS quartiles do not differ in age, CD4 T cell counts, and plasma viral RNA loads.** p values are from one-way ANOVA.

Figure S2

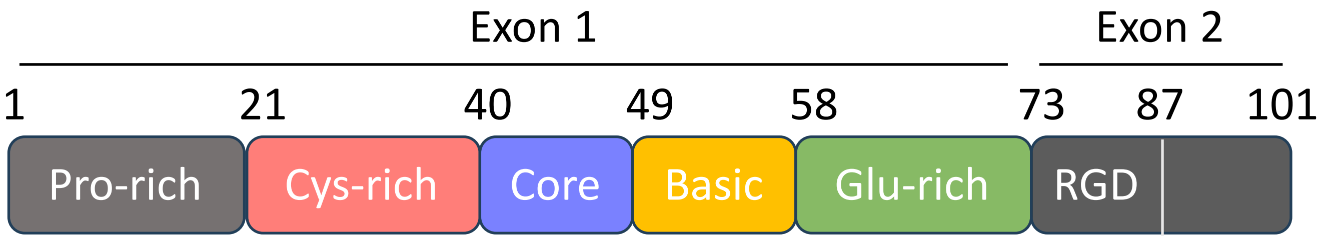


1 86

NL4.3: MEPVDPRLEPWKHPGSQPKTACTNCYCKKCCFHCQVCFMTKALGISYGRKKRRQRRRAHQNSQTHQASLSKQPTSQSRGDPTGPKE 101

ConsB: MEPVDPRLEPWKHPGSQPKTACTNCYCKKCCFHCQVCFITKGLGISYGRKKRRQRRRAPQDSQTHQVSLSKQPASQPRGDPTGPKE*SKKKVERETETDPVDQ*

Pool 1
MEPVDPRLEPWKHPG
DPRLEPWKHPGSQPK
EPWKHPGSQPKTACT

Pool 2
HPGSQPKTACTNCYC
QPKTACTNCYCKKCC
ACTNCYCKKCCFHCQ
CYCKKCCFHCQVCFI

Pool 3
KCCFHCQVCFITK
HCQVCFITKGLGISY
CFITKGLGISYGRKK
KGLGISYGRKKRRQR

Pool 4
ISYGRKKRRQRRRAP
RKKRRQRRRAPQDSQ
RQRRRAPQDSQTHQV

Pool 5
RAPQDSQTHQVSLSK
DSQTHQVSLSKQPAS
HQVSLSKQPASQPRG
LSKQPASQPRGDPTG
PASQPRGDPTGPKES

Pool 6
PRGDPTGPKESKKKV
PTGPKESKKKVERET
KESKKKVERETETDP
KKVERETETDPVDQ

**Supplemental Figure S2. Amino acid sequences of Tat peptides tested in this study.** Overlapping peptides encompassing 101 amino acids of consensus B HIV-1 Tat were divided into six pools that represent the six regions of Tat: proline-rich, cysteine-rich, core, basic, glutamic acid-rich, and RDG plus the C-terminus. Comparison of consensus B and NL4.3 sequences reveals distinct amino acids that are denoted in red and the missing C-terminal region in the NL4.3 sequence (Genbank: AF324493.2).

Figure S3

A

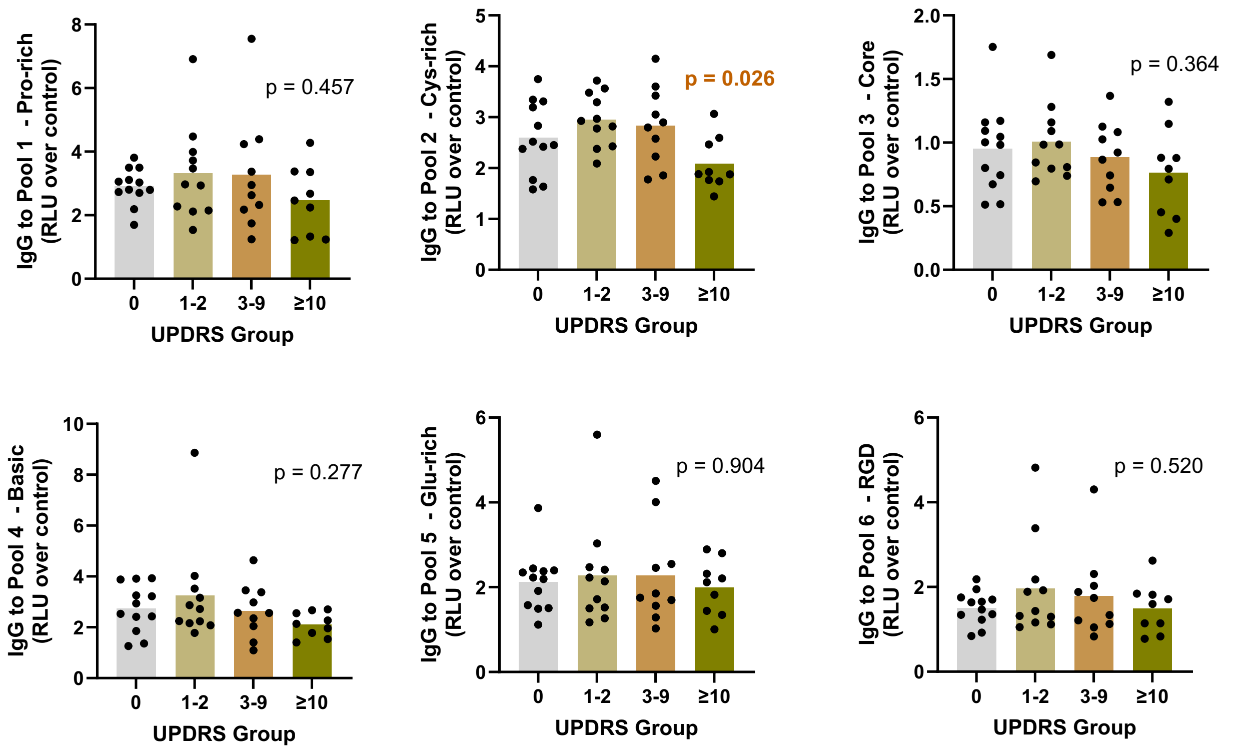


B

| IgG to Tat peptides | Global T ≥40 vs. <40 p value |
|---|---|
| Pool 1 | 0.235 |
| Pool 2 | 0.448 |
| Pool 3 | 0.550 |
| Pool 4 | 0.360 |
| Pool 5 | 0.610 |
| Pool 6 | 0.587 |

**Supplemental Figure S3. IgG levels against the six Tat regions in PWH with different UPDRS or Global T-score groups.**
A. Relative levels of plasma IgG antibodies against six pools of peptides representing the different regions of Tat in PWH with varying UPDRS scores. The 42 PWH were grouped into four groups based on their UPDRS scores. p values from ANOVA are shown.
B. Comparison of IgG levels against the six peptide pools in PWH with Global T-scores of ≥40 (normal) or <40 (deficient). p values from Mann-Whitney test are shown.

Figure S4

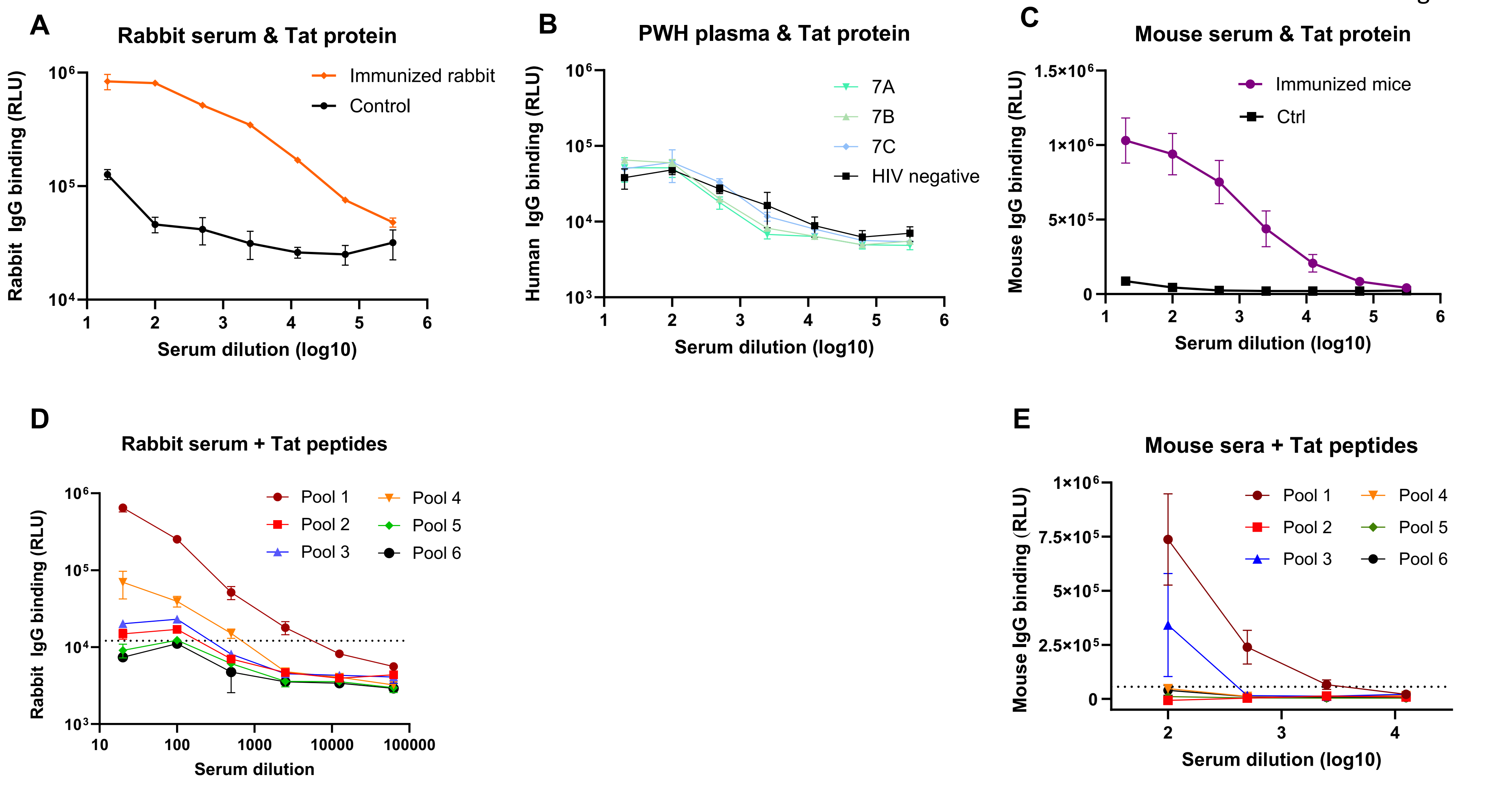

A
Rabbit serum & Tat protein
Immunized rabbit
Control
Rabbit IgG binding (RLU)
Serum dilution (log10)
B
PWH plasma & Tat protein
7A
7B
7C
HIV negative
Human IgG binding (RLU)
Serum dilution (log10)
C
Mouse serum & Tat protein
Immunized mice
Ctrl
Mouse IgG binding (RLU)
Serum dilution (log10)
D
Rabbit serum + Tat peptides
Pool 1
Pool 2
Pool 3
Pool 4
Pool 5
Pool 6
Rabbit IgG binding (RLU)
Serum dilution
E
Mouse sera + Tat peptides
Pool 1
Pool 2
Pool 3
Pool 4
Pool 5
Pool 6
Mouse IgG binding (RLU)
Serum dilution (log10)

**Supplemental Figure S4. Titration of anti-Tat IgG antibodies in immunized rabbits and mice vs. PWH.**

A-C. Reactivity of IgG antibodies against Tat protein in titrated samples from immune vs. control rabbits (A), PWH vs. PWOH (B), and immunized vs. control mice (C).

D-E. Reactivity of IgG antibodies in titrated sera from immunized rabbits (D) and mice (E) against six pools of Tat peptides; areas under the titration curves are calculated and shown in Figures 4D and 4F, respectively. Dotted lines: unimmunized controls.

RLU: relative luminescence unit.
